# Clinical Patient-Relevant Outcome Domains for Persistent Spinal Pain Syndrome—A Scoping Review and Expert Panels

**DOI:** 10.3390/jcm13071975

**Published:** 2024-03-28

**Authors:** Ferdinand Bastiaens, Jessica T. Wegener, Raymond W. J. G. Ostelo, Bert-Kristian W. P. van Roosendaal, Kris C. P. Vissers, Miranda L. van Hooff

**Affiliations:** 1 Department of Research, Sint Maartenskliniek, 9500 GM Nijmegen, The Netherlands; 2 Department of Anesthesiology, Pain, and Palliative Medicine, Radboud University Medical Center, Geert Grooteplein Zuid 10, 6525 GA Nijmegen, The Netherlands; 3 Department of Anesthesiology and Pain Medicine, Sint Maartenskliniek, 9500 GM Nijmegen, The Netherlands; 4 Department of Health Sciences, Faculty of Science and Amsterdam Movement Science Research Institute, Vrije Universiteit, Van der Boechorststraat 7, 1081 BT Amsterdam, The Netherlands; 5 Department of Epidemiology and Data Science, Amsterdam University Medical Centre, Vrije Universiteit, Meibergdreef 9, 1105 AZ Amsterdam, The Netherlands; 6 Department of Orthopedics, Radboud University Medical Center, Geert Grooteplein Zuid 10, 6525 GA Nijmegen, The Netherlands

**Keywords:** persistent spinal pain syndrome, scoping review, outcome domains, patient participation, expert panel

## Abstract

Large variation exists in the monitoring of clinical outcome domains in patients with persistent spinal pain syndrome (PSPS). Furthermore, it is unclear which outcome domains are important from the PSPS patient’s perspective. The study objectives were to identify patient-relevant outcome domains for PSPS and to establish a PSPS outcomes framework. PubMed, CINAHL, Cochrane, and EMBASE were searched to identify studies reporting views or preferences of PSPS patients on outcome domains. The Arksey and O’Malley framework was followed to identify outcome domains. An expert panel rated the domains based on the importance for PSPS patients they have treated. A framework of relevant outcome domains was established using the selected outcome domains by the expert panel. No studies were found for PSPS type 1. Five studies with 77 PSPS type 2 patients were included for further analysis. Fourteen outcome domains were identified. An expert panel, including 27 clinical experts, reached consensus on the domains pain, daily activities, perspective of life, social participation, mobility, mood, self-reliance, and sleep. Eleven domains were included in the PSPS type 2 outcomes framework. This framework is illustrative of a more holistic perspective and should be used to improve the evaluation of care for PSPS type 2 patients. Further research is needed on the prioritization of relevant outcome domains.

## 1. Introduction

Persistent spinal pain syndrome (PSPS) encompasses a diversity of clinical symptoms. These include chronic or recurrent pain of spinal origin, paresthesia, numbness, stiffness, muscle spasms, and weakness, most commonly situated in the lumbosacral region [1,2,3,4]. Spinal surgery may have occurred (PSPS type 2, formerly known as failed back surgery syndrome (FBSS)) or not (PSPS type 1) [4]. PSPS patients commonly suffer from severe complaints [5], impacting their ability to work [6] and diminishing their quality of life [7]. A multitude of interventions are frequently offered to PSPS patients in primary care and dedicated pain centers, ranging from conservative therapy to invasive pain treatments [8,9,10]. 

Clinical outcome domains are defined as concepts to be measured in terms of a further specification of an aspect of health [11]. Ideally, there should be a consensus-based set of outcomes that can be monitored over time, reported in research trials, and in daily clinical practice of a specific clinical area [12]. Although PSPS patients often share epidemiological, demographic, and phenotypical characteristics, a large variation exists in the monitoring of clinical outcome domains [13]. This is partly because of the possible refractory character of this syndrome and the various clinical approaches and care pathways provided by different medical specialties who are involved in the management of PSPS patients. Furthermore, the tools used to measure the properties of these outcome domains vary largely [14,15,16]. These inconsistencies impede large-scale evaluations and the ability to make informed decisions about healthcare [17]. 

A standardized set of outcomes that focuses on biomedical, psychosocial, and behavioral domains is needed to map the health status of chronic pain patients [18]. In general, classification models such as the International Classification of Diseases (ICD-11) and the International Classification of Functioning, Disability and Health (ICF) aim to identify the right patient populations and emphasize a broader view on health, where health encompasses more than the absence of a disease [19,20]. In addition, conversational tools such as the Positive Health Model focus on the multidimensional exploration of patient preferences in the clinical setting [21]. 

There are also initiatives that recommend multidimensional outcome domains for (non-specific) low back pain [22,23]. However, due to the chronic and multi-dimensional nature of PSPS, these recommendations may not be appropriate for PSPS patients [10,24]. In addition, there are recommendations on outcome domains in chronic pain trials, as well as a consensus statement on outcome domains for PSPS type 2 patients utilizing a multidisciplinary team approach [15,25]. However, these recommendations are treatment related and based on the perspectives of clinical and scientific experts. Overall, it is important that the patient’s perspective on outcome domains is more involved in these clinical outcome sets to ensure the clinical relevance [26]. 

The clinical relevance of measured outcome domains is important in addressing the healthcare needs of patients and facilitate the process of shared decision making [27,28,29,30,31]. Due to the chronic nature of PSPS and multidimensional limitations in daily life for PSPS patients, it is important to consider the value of different domains from a patient’s perspective. Hence, a more multidimensional evaluation is necessary to determine which outcome domains are deemed important from the perspective of PSPS patients. The primary objective of this study is to identify outcome domains from the perspective of patients with PSPS (patient-relevant outcome domains). Additionally, we aim to link the identified outcome domains to items of the ICF model. 

## 2. Materials and Methods

This study is the first part of a research project to identify a shortlist of patient-relevant outcome domains. The research project follows an iterative design in accordance with the core outcome set process described in the COMET Handbook [17]. A scoping review of the literature is performed to identify existing evidence, followed by a consensus process with a panel of clinical and research experts to elicit views about the outcome domains. In a subsequent study, focus groups will be held with PSPS patients to weigh and prioritize (and possibly expand the list of) the identified outcome domains of the current publication.

In this study, a scoping literature review was performed to explore the perspectives and preferences of PSPS patients on important outcome domains. The framework of Arksey and O’Malley was followed [26]. This framework provides a comprehensive foundation for scoping review methodology comprising five stages: (1) identifying the research question; (2) identifying relevant studies; (3) study selection; (4) charting the data; and (5) collating, summarizing, and reporting the results. The list of outcome domains was evaluated by (6) consulting expert panels to determine a framework of relevant outcome domains. The study is performed and reported according to the Preferred Reporting Items for Systematic Reviews and Meta-Analysis (PRISMA) statement for scoping reviews [32].

### 2.1. Identifying the Research Question

The aim of the scoping review was to identify patient-relevant outcome domains for PSPS patients that could be used by PSPS patients to weigh and prioritize the identified domains. The following research questions were formulated: 1. Which outcome domains are deemed relevant for the general health of PSPS patients? 2. Can the identified outcome domains be linked to the items of the ICF model and used to create a PSPS outcome-framework. 

### 2.2. Identifying Relevant Studies

The literature search using PubMed, CINAHL, Embase, and Cochrane Library was performed in November 2023. The search strategy was set up with the aid of an information specialist and consisted of keywords, subject headings, and free-text words. The search string was built upon a combination of the patient populations (e.g., chronic pain), possible interventions (e.g., pain management), and outcomes (e.g., patient participation). The complete search string is shown in Supplementary Materials Section S1. Studies found through the search results were imported and managed in Rayyan QCRI [33].

### 2.3. Study Selection

Studies focusing on PSPS patients encompassing a diversity of clinical symptoms were eligible for inclusion. These include chronic or recurrent pain of spinal origin, paresthesia, numbness, stiffness, muscle spasms, and weakness, most commonly situated in the lumbosacral region and [1,2,3,4]. Spinal surgery may have occurred (PSPS type 2, including previous diagnoses such as FBSS and post-laminectomy syndrome) or not (PSPS type 1) [4]. Furthermore, studies had to contain views or preferences of PSPS patients on outcome domains. Both qualitative and quantitative studies were eligible for inclusion. Case reports, animal studies, in vitro studies, biomechanical studies, simulation studies, and literature reviews were excluded. Non-English language studies, conference abstracts, and study protocols were excluded as well. In Table 1, an overview is presented of the selection criteria following the participants/population, intervention, comparator and outcome model (PICO). 

After checking for duplicates, all the studies of the initial search were screened based on title and abstract. Included studies were checked on full text-availability. All full-text studies were then subjugated to full-text screening. Both screening processes were conducted separately by two reviewers (F.B. and B.R.). In case of disagreements, the reviewers discussed the study until consensus was reached. 

### 2.4. Charting the Data and Collating, Summarizing and Reporting the Results

The following categories of information were extracted from included studies: author(s), year of publication, objective(s), study design, setting, country, study population, and sample size. In quantitative studies, identified outcome domains and their rationale were charted and compiled into a list. Qualitative studies were analyzed through theoretical thematic analysis [34,35]. The first step was familiarization of the collected data. Secondly, all key themes were identified in order to further develop the framework. Thirdly, data were indexed in textual form by coding the relevant information from the studies. Fourthly, data were linked to the relevant part of the thematic framework in concordance with the ICF rules [19,36,37]. Outcome domains recurring in multiple studies were considered as patient-relevant outcome domains and included for further evaluation by the expert panels in order to establish a PSPS outcomes framework.

### 2.5. Expert Panel Consultation

The list of outcome domains linked to the ICF models was presented to an expert panel. The expert panel consultation consisted of a two-round online questionnaire, followed by a consensus meeting. The experts were medical specialists experienced in treating PSPS patients and were recruited from the Orthopedics and Chronic Pain departments of the Sint Maartenskliniek Nijmegen and Radboud University Medical Center Nijmegen. The expert panel was asked to complete a questionnaire in which they were asked to rate the domains based on the importance for PSPS patients they have treated. In the first round, experts were asked to rate each outcome domain using the Grading of Recommendations Assessment, Development and Evaluation (GRADE) scale, a nine-point scale that is commonly divided into three categories for Core Outcome Set projects: not important (1–3), important but not critical (4–6), and critically important (7–9) [38]. A free-text option was also included to add comments or suggestions for additional outcomes. After the first round, the results of the first round were discussed in a consensus meeting by participating experts. Descriptive statistics (e.g., median and interquartile range (IQR)) were used to analyze the results of both rounds. 

In the first round, 18 experts from the chronic pain department (chronic pain expert panel) and nine experts from the orthopedic department (orthopedic expert panel) participated. In total, 11 experts from the chronic pain department also participated in the second round. The chronic pain expert panel consisted of seven anesthesiologists, six neurosurgeons, and five nursing specialists, whereas the orthopedic panel consisted of four orthopedic spinal surgeons, four general orthopedists, and one spine orthopedist.

Defining consensus for inclusion of an outcome in the shortlist was based on the systematic review on consensus in Delphi studies by Diamond et al. (2014) [39]. Consensus was defined a priori as ≥75% of the participants in all stakeholder groups rating the outcome as critically important (GRADE score = 7–9) [39]. Consensus for exclusion of an outcome from the shortlist was defined as 50% or less of respondents in all stakeholder groups rating the outcome as critically important [40]. Added suggestions were reviewed by the research team and, if appropriate, included as an outcome domain in the second round. 

Prior to the second round, an overview of the included and excluded domains from the first round was shown and discussed with the experts. The experts were asked to give a new GRADE rating. Inclusion/exclusion of outcome domains was based on the afore-mentioned consensus measures. After the second round, outcome domains that did not meet either measure were assessed by the research team. A framework of relevant outcome domains was established using the ICF model, in which the outcome domains selected by the two rounds of experts were linked to items of the ICF classification [19,36,37]. The outcome domains were linked to the most precise ICF level of classification (or category). The ICF categories ‘other specified’ and ‘unspecified’ were avoided in the linking process. The main researcher (FB) performed the initial linking process, which was discussed the main research team (JW, MH, JV, and KV) in order to reach consensus for the final linkage decisions. Outcome domains that could not be classified in the ICF were labeled as “not covered”, and those that were not precise enough were labeled as “not definable”, apart from outcome domains that were considered as personal factors.

## 3. Results

### 3.1. Study Selection

**Figure 1:**
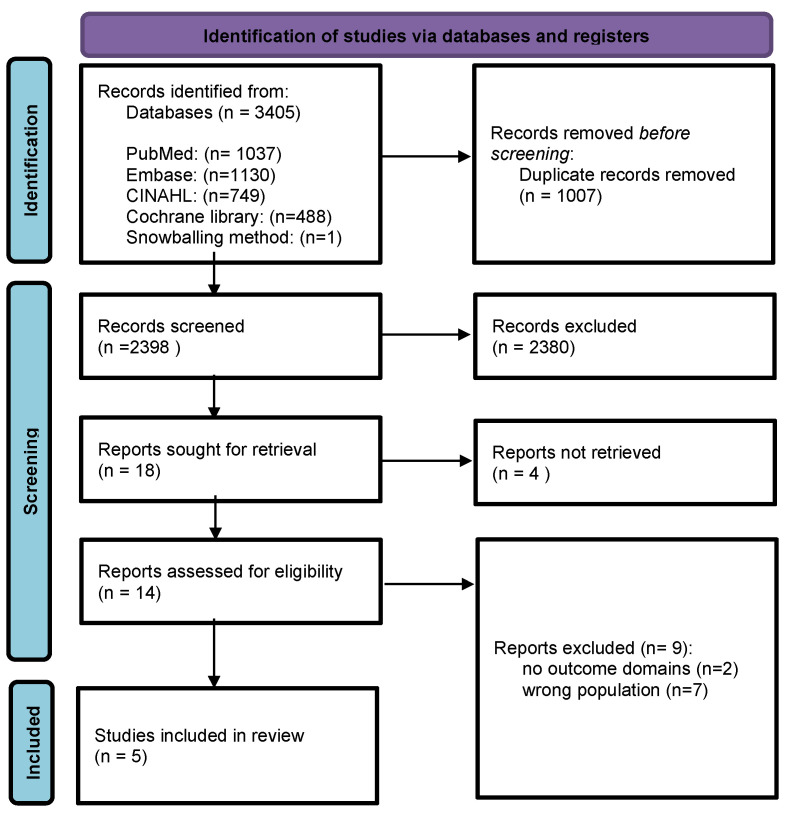
PRISMA flowchart of the study selection and eligibility process.

The databases yielded 3405 potentially relevant published studies, of which 2398 studies remained after the duplication check. After screening the titles and abstracts, 18 studies remained. During the full-text availability check, 4 studies could not be retrieved. Of the 14 studies that underwent a full-text screening, 9 were excluded due to wrong populations (e.g., non-specific low-back pain, spinal cord injury, fibromyalgia, diabetes, etc.) and/or absence of reported patient perspectives on outcome domains [41,42,43,44,45,46,47,48,49]. A final number of 5 studies were included for further analysis. The screening process is shown in the study flow diagram (Figure 1).

### 3.2. Study Characteristics

Included studies were conducted in four different European countries. All studies were qualitative single-center studies conducted in a hospital setting. Sample sizes in the studies ranged from 12 to 20 participants, with 77 participants in total. All the included study populations are classified as PSPS type 2. Specifically, four of the included studies focused on spinal cord stimulation (SCS) in PSPS type 2 patients either treated with SCS or being considered candidates for SCS treatment. Three studies reported to have no conflicts of interest, and one study lacked a report on conflicts of interest. One study was funded by a medical company, while another study was supported by a medical company. An overview of the characteristics is shown in Table 2. 

### 3.3. Patient-Relevant Outcome Domains

Based on the data chart, fourteen patient-relevant outcome domains were identified. The outcome domains pain and mobility were identified in all the included studies, whereas pain medication, daily activities, work, social participation, leisure activities, and mood were identified in four studies. In three studies, the outcome domains coping strategy, sleep, and energy were reported. The outcome domains of acceptance, perspective of life, and self-reliance were noted twice in the included studies. An overview of the characteristics is shown in Table 3. Thirteen outcome domains were identified in only a single study and therefore not included. A qualitative overview of the identified outcome domains can be seen in Supplementary Materials Section S2.

### 3.4. Expert Panel Consultation

#### 3.4.1. First Consensus Round

After the first round, the following domains reached consensus for inclusion: pain, sleep, daily activities, perspective of life, social participation, mood, and self-reliance. The domains coping strategy, work, and acceptance were excluded from the framework. While discussing the results of the first round, the participating experts noted that coping strategy and acceptance were relevant domains for patients, but only at a later stage in their care journey. In addition, work was considered less relevant due to the relatively large proportion of PSPS patients who are retired or about to retire or are on long-term disability. A complete overview of the results from the first round is shown in Table 4. 

#### 3.4.2. Second Consensus Round

The outcomes suggested by panelists secondary gain and external perception were included in the second round. However, both were subsequently excluded. A complete overview of suggested outcomes is shown in Supplementary Materials Section S3. The outcome domain mobility was included based on consensus. An overview of the results from the second round is shown in Table 5.

**Figure 2:**
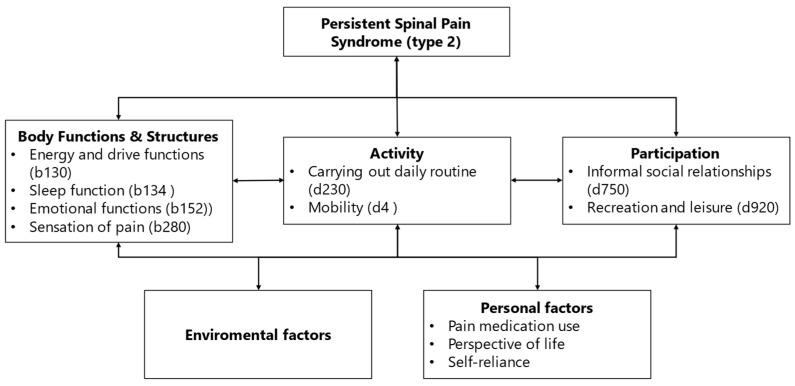
The PSPS type 2 outcomes framework of patient-relevant outcome domains for PSPS type 2 with ICF classifications.

The remaining outcome domains (pain medication use, leisure activities and energy) were included in the final framework after a discussion among the research team, alongside the previously included domains from the first round. A complete overview of the results of the second round is shown in Table 5. The final framework, the PSPS type 2 outcomes framework, was determined by linking the included outcome domains to items of the ICF model (Figure 2). 

## 4. Discussion

With this scoping review, we aimed to identify relevant outcome domains for PSPS from the patient perspective (patient-relevant outcome domains). Five studies (77 patients) were included in this scoping review. Out of these studies, 14 patient-relevant outcome domains were identified. In two expert panel rounds, consisting of 27 experts, the outcome domains were rated on their importance until consensus was reached. The following 11 outcome domains reached consensus and were included in the PSPS type 2 outcomes framework and based on the ICF classification: pain, daily activities, perspective of life, social participation, sleep, mobility, mood, pain medication, leisure activities, energy, and self-reliance (Figure 2). 

### 4.1. Comparison with Other Studies

The identified outcome domains in the PSPS type 2 outcomes framework comprise an expansive set, illustrative of a holistic perspective on PSPS. Several outcome sets for chronic (low back) pain exist. For example, the International Consortium for Health Outcomes Measurement (ICHOM) has developed a set of Patient-Centered Outcome Measures for Low Back Pain [22]. Additionally, the Initiative on Methods, Measurement, and Pain Assessment in Clinical Trials (IMMPACT) recommends a core set of outcome measures in chronic pain trails [14]. Comparing the ICHOM-LBP set with our framework, a notable difference is the more generalized nature of the domains (such as health-related quality of life and disability). Furthermore, the ICHOM set contains work status, while this outcome domain is excluded from the framework in the expert panels. This might be related to the relatively high percentage of retirees and work-related disability among patients with PSPS, which was mentioned in the expert panels [55].

In contrast to our developed outcomes framework, the IMMPACT core outcome set contains some intervention-related aspects, such as adverse events and treatment satisfaction. In addition to pain intensity, IMMPACT recommends emotional functioning as an outcome domain, which includes both depression and mood in general. Although patients in Goudman et al., (2020) specifically mention avoiding depression, it is not discussed in the other included studies of our scoping review [51]. This may be due to a relative lack of focus on the clinical diagnosis of depression in chronic pain patients, where more attention is paid to the impact of the complaints on their lives, such as mood and perspective of life.

Furthermore, both the recommended outcome sets of ICHOM and IMMPACT are linked to PROMs. Some PROMs, such as the Short-Form Health Survey (SF-36), have a broad and generalized character, in which multiple outcome domains are queried. However, this makes is difficult to monitor specific outcome domains, such as sleep. Moreover, ICHOM and IMMPACT recommend different PROMs for similar outcome domains, apart from the NPRS for pain. In this scoping review, we did not consider measurement instruments, such as PROMs. It is unclear which measurement instruments (e.g., PROMs) are adequate, in terms of measurement properties to coherently capture the identified patient perspectives and values. International consensus is needed on core outcome domains and corresponding outcome measures for chronic low back pain and specifically for PSPS.

### 4.2. Strengths and Limitations

To our knowledge, this is the first literature review focusing on the PSPS patient perspective on outcome domains. The qualitative nature of the included studies is of great added value by providing insight into the values, beliefs, and experiences of PSPS patients. It resulted in a multidimensional and clinically relevant set of outcome domains. Furthermore, the additional expert panels contributed to the existing data from the review. The experts were able to draw on their extensive experiences with a large group of PSPS patients. By including different types of healthcare disciplines involved in the diagnostic process and care for PSPS patients, we ensured the expertise on the needs of PSPS patients in different phases of their hospital care journey.

Another strength of this study is that the domains in the PSPS type 2 outcomes framework are linked to items of the ICF model. By linking the framework to the ICF, the framework consists of uniform and internationally accepted definitions. The framework is therefore very useful in various clinical settings, as well as future research, e.g., into adequate measuring instruments.

This review also has some limitations. First, a small number of relevant studies from Northern and Western Europe were included. The lack of relevant studies in the literature might be due to the specific inclusion criteria for PSPS patients, as well as the criteria for outcome domains. The relative cultural homogeneity might be of limiting influence, in particular when related to the personal factors in the PSPS type 2 framework. Second, the included studies consisted of relatively small sample sizes. This might be related to the qualitative nature of the included studies. Nonetheless, our goal is to follow up our research with a focus group study to expand and deepen the available data on this topic through an emphasis on prioritization of the relevant outcome domains. Third, the generalizability of the results seems limited due to the absence of type 1 PSPS patients. This could be explained by a recent change in terminology. While the term failed back surgery syndrome (FBSS) can be converted to PSPS type 2, it is unclear which patients can be classified as PSPS type 1. It is questionable whether the views on outcome domains of type 1 PSPS patients differ since the distinction between the two groups is based on a difference in (surgical) history rather than a difference in symptoms [56]. However, the outcome assessment of type 1 and type 2 PSPS patients will likely differ as a result of different treatment options, such as SCS.

Finally, the majority of included studies was skewed towards either PSPS patients treated with SCS or SCS candidates. In general, SCS has been the most frequently studied treatment method for type 2 PSPS patients [8,13]. However, PSPS patients treated with SCS might not be reflective of the general PSPS population. More research is needed on relevant outcome domains for PSPS patients who benefit from non-invasive and minimally invasive treatments.

### 4.3. Implications

The PSPS type 2 outcomes framework (Figure 2) shows a detailed and multidimensional set of relevant outcome domains for PSPS patients. It should be taken into account that the excluded domains acceptance, work, and coping strategy may also be relevant for subgroups within the PSPS population. This partly depends on the phase of the care process in which the patient is. When evaluating care, it is important that there is also room for the personal needs and goals of the patients [57].

A possible way to evaluate the multidimensional and personal picture in a clinical setting is through the Positive Health Model [21]. Although this model is used as a conversation tool for exploring patient-relevant outcome domains, one can use it to combine the complexity associated with chronic pain with setting patient-centered goals. This can also support the process of shared decision making. It should also be considered that patients themselves usually do not know in advance what to expect regarding the effect of a treatment. Therefore, it is necessary to systematically compare PROMs and patient-reported experience measures (PREMs). The expectations of the care provider about the possible effect of a treatment should also be mentioned and explored.

In summary, we recommend using the PSPS type 2 outcomes framework with patient -relevant outcome domains (Figure 2) to improve the evaluation of care for PSPS patients by evaluating healthcare multidimensionally and placing a relatively smaller focus on pain. This also applies to insurance companies and healthcare institutions that want to have high impact clinical evaluation tools to observe real, stable, and relevant long-term clinical outcomes. The framework is complementary to initiatives such as the holistic treatment response for SCS [58]. These evaluation techniques would be further substantiated with clinical outcome domains prioritized by PSPS patients.

## 5. Conclusions

With our scoping review and expert panels, we have identified the following 11 patient-relevant outcome domains for PSPS type 2: (1) pain, (2) sleep, (3) daily activities, (4) mobility, (5) energy, (6) mood, (7) perspective of life, (8) social participation, (9) self-reliance, (10) leisure activities, and (11) pain medication use. The outcome domains comprise an expansive set illustrative of a more holistic approach to PSPS type 2. An absence of the literature regarding the perspective of PSPS type 1 patients limited further analysis. The PSPS type 2 outcomes framework with ICF-linked domains should be used to improve the evaluation of care for PSPS type 2 patients by evaluating healthcare multidimensionally. Further research is needed on the prioritization of the relevant outcome domains for PSPS patients.

## Figures and Tables

**Table 1:** PICO for the scoping review.

Category	Selection Criteria
Participants/population	Adult (≥18 years) PSPS patients who present back and/or leg pain and irrespective of whether they have undergone prior back surgery or not. This includes study samples with an FBSS diagnosis.
Intervention	Not applicable
Comparator	Not applicable
Outcome	Views or preferences of PSPS patients on outcome domains.

**Table 2:** Study characteristics of the included studies.

Author (Year)	Objective(s)	Design	Country (Setting)	Study Population	Sample Size	Conflict of Interest & Funding
Abbot et al. (2011) [50]	To describe within the context of the ICF, patients’ experiences post-lumber fusion regarding back problems, recovery, and expectations of rehabilitation.	(Semi-structured) interview study	Sweden (Hospital: Orthopedic department)	CLBP patients post lumbar-fusion (PSPS type 2)	20	No conflicts of interest statement. This study was funded by a research grant obtained from the Health Care Sciences Postgraduate School, Karolinska Institute.
Goudman et al. (2020) [51]	Explore if applying goal setting, as a form of patient empowerment, in potential candidates for SCS may further improve the outcome of SCS.	(Semi-structured) interview study	Belgium (Hospital: Neurosurgery department)	SCS candidates with FBSS or FNSS (PSPS type 2)	15	Authors have no conflicts of interest to declare. Study was supported by Medtronic.
Hamm-Faber et al. (2020) [52]	To explore perspectives on personal health and quality of life in FBSS patients concerning their physical, psychological and spiritual well-being prior to receiving an SCS system.	(Semi-structured) interview study	Netherlands (Hospital: Pain medicine department)	SCS candidates with FBSS (PSPS type 2)	17	No competing interests. Study received no external funding.
Ryan et al. (2019) [53]	To explore the experience of SCS for patients with FBSS.	(Semi-structured) interview study	United Kingdom (Hospital: Pain clinic)	SCS patients with FBSS (PSPS type 2)	12	Dr. Cormac G Ryan and Professor Denis J. Martin are named inventors on a patent application for a novel device that delivers sensory discrimination training. The device could be used in the treatment of people with chronic pain. The remaining authors have no conflicts of interest to declare. Funded by Medtronic.
Witkam et al. (2021) [54]	To qualitatively and quantitatively map the FBSS patients’ experiences with SCS and the effects of SCS on low back pain caused by FBSS.	Qualitatively driven mixed method analysis	Netherlands (Hospital: Anaesthesiology department)	SCS patients with FBSS (PSPS type 2)	13	The authors reported no conflict of interest. No financial support.

CLBP: chronic low back pain

**Table 3:** Included outcome domains.

	Abbot et al. (2011) [50]	Goudman et al. (2020) [51]	Hamm-Faber et al. (2020) [52]	Ryan et al. (2019) [53]	Witkam (2021) [54]
Pain	X	X	X	X	X
2. Mobility	X	X	X	X	X
3. Work	X	X	X		X
4. Social participation	X	X	X		X
5. Mood	X	X		X	X
6. Pain medication use	X		X	X	X
7. Daily activities	X		X	X	X
8. Leisure activities/hobbies	X	X	X		X
9. Coping strategy	X		X		X
10. Energy	X		X		X
11. Sleep			X	X	X
12. Acceptance			X		X
13. Perspective of life			X		X
14. Self-reliance			X		X

X indicates that the outcome domain is described in the specific study.

**Table 4:** GRADE results from the first round of expert panels.

Outcome Domain	Score 7–9 (n Panelists)	Score 4–6 (n Panelists)	Score 1–3 (n Panelists)	Median (IQR) *	Consensus ^#^
Pain	26	0	1	8 (8–9)	≥75%
Coping Strategy	8	18	1	6 (5–7)	<50%
Pain Medication Use	19	8	0	7 (6–8)	50–75%
Sleep	21	6	0	8 (7–8)	≥75%
Daily Activities	22	5	0	8 (7–8)	≥75%
Mobility	20	7	0	7 (6.5–8)	50–75%
Work	11	16	0	6 (5–7)	<50%
Acceptance	10	13	4	6 (4.5–7)	<50%
Perspective of life	23	4	0	7 (7–8)	≥75%
Social participation	24	3	0	7 (7–8)	≥75%
Mood	21	6	0	7 (7–8)	≥75%
Self-Reliance	21	6	0	7 (6–8)	≥75%
Leisure Activities	19	8	0	7 (7–8)	50–75%
Energy	16	10	1	7 (6–8)	50–75%

* IQR interquartile range; ^#^: <50% = excluded, 50–75% = subject to further discussion, ≥75% = included.

**Table 5:** GRADE results from the second round of expert panels.

Outcome Domain	Score 7–9 (n Panelists)	Score 4–6 (n Panelists)	Score 1–3 (n Panelists)	Median (IQR) *	Consensus ^#^
Pain Medication Use	6	5	0	7 (6–7.5)	50–75%
Mobility	10	1	0	7 (7–8)	≥75%
Leisure Activities	8	3	0	7 (6.5–7.5)	50–75%
Energy	7	4	0	7 (6–7)	50–75%
External Perception	4	6	1	6 (5–7)	<50%
Secondary gain	1	5	5	4 (2.5–5)	<50%

* IQR interquartile range; ^#^: <50% = excluded, 50–75% = subject to further discussion, ≥75% = included.

## Data Availability

The raw data supporting the conclusions of this article will be made available by the authors on request.

## Supplementary Materials

The following supporting information can be downloaded at: https://www.mdpi.com/article/10.3390/jcm13071975/s1, Section S1: Search string (PubMed); Section S2: Qualitative overview of outcome domains from included studies.; Section S3: Expert panel suggestions and discussion (translated from Dutch).

## References

[1] Merskey H.E. (1986). Classification of chronic pain: Descriptions of chronic pain syndromes and definitions of pain terms. Pain.

[2] Follett K.A., Dirks B.A. (1993). Etiology and evaluation of the failed back surgery syndrome. Neurosurg. Q..

[3] Leveque J.C., Villavicencio A.T., Bulsara K.R., Rubin L., Gorecki J.P. (2001). Spinal cord stimulation for failed back surgery syndrome. Neuromodulation.

[4] Christelis N., Simpson B., Russo M., Stanton-Hicks M., Barolat G., Thomson S., Schug S., Baron R., Buchser E., Carr D.B. (2021). Persistent Spinal Pain Syndrome: A Proposal for Failed Back Surgery Syndrome and ICD-11. Pain Med..

[5] Yorimitsu E., Chiba K., Toyama Y., Hirabayashi K. (2001). Long-term outcomes of standard discectomy for lumbar disc herniation: A follow-up study of more than 10 years. Spine.

[6] Kumar K., North R., Taylor R., Sculpher M., Van den Abeele C., Gehring M., Jacques L., Eldabe S., Meglio M., Molet J. (2005). Spinal Cord Stimulation vs. Conventional Medical Management: A Prospective, Randomized, Controlled, Multicenter Study of Patients with Failed Back Surgery Syndrome (PROCESS Study). Neuromodulation.

[7] Manca A., Eldabe S., Buchser E., Kumar K., Taylor R.S. (2010). Relationship between health-related quality of life, pain, and functional disability in neuropathic pain patients with failed back surgery syndrome. Value Health.

[8] Amirdelfan K., Webster L., Poree L., Sukul V., McRoberts P. (2017). Treatment Options for Failed back Surgery Syndrome Patients with Refractory Chronic Pain: An Evidence Based Approach. Spine.

[9] Chan C.W., Peng P. (2011). Failed back surgery syndrome. Pain Med..

[10] Sebaaly A., Lahoud M.J., Rizkallah M., Kreichati G., Kharrat K. (2018). Etiology, evaluation, and treatment of failed back surgery syndrome. Asian Spine J..

[11] Boers M., Kirwan J.R., Wells G., Beaton D., Gossec L., d’Agostino M.A., Conaghan P.G., Bingham C.O., Brooks P., Landewé R. (2014). Developing core outcome measurement sets for clinical trials: OMERACT filter 2.0. J. Clin. Epidemiol..

[12] Williamson P.R., Altman D.G., Blazeby J.M., Clarke M., Devane D., Gargon E., Tugwell P. (2012). Developing core outcome sets for clinical trials: Issues to consider. Trials.

[13] Cho J.H., Lee J.H., Song K.S., Hong J.Y., Joo Y.S., Lee D.H., Hwang C.J., Lee C.S. (2017). Treatment Outcomes for Patients with Failed Back Surgery. Pain Physician.

[14] Dworkin R.H., Turk D.C., Farrar J.T., Haythornthwaite J.A., Jensen M.P., Katz N.P., Kerns R.D., Stucki G., Allen R.R., Bellamy N. (2005). Core outcome measures for chronic pain clinical trials: IMMPACT recommendations. Pain.

[15] Rigoard P., Gatzinsky K., Deneuville J.P., Duyvendak W., Naiditch N., Van Buyten J.P., Eldabe S. (2019). Pain Research and Management, 2019. Optimizing the management and outcomes of failed back surgery syndrome: A consensus statement on definition and outlines for patient assessment. Pain Res. Manag..

[16] Clancy C., Quinn A., Wilson F. (2017). The aetiologies of failed back surgery syndrome: A systematic review. J. Back Musculoskelet. Rehabil..

[17] Williamson P.R., Altman D.G., Bagley H., Barnes K.L., Blazeby J.M., Brookes S.T., Clarke M., Gargon E., Gorst S., Harman N. (2017). The COMET handbook: Version 1.0. Trials.

[18] Dansie E.J., Turk D.C. (2013). Assessment of patients with chronic pain. Br. J. Anaesth..

[19] World Health Organization (2001). International Classification of Functioning, Disability, and Health.

[20] Treede R.D., Rief W., Barke A., Aziz Q., Bennett M.I., Benoliel R., Cohen M., Evers S., Finnerup N.B., First M.B. (2019). Chronic pain as a symptom or a disease: The IASP classification of chronic pain for the international classification of diseases (ICD-11). Pain.

[21] Huber M., van Vliet M., Giezenberg M., Winkens B., Heerkens Y., Dagnelie P.C., Knottnerus J.A. (2016). Towards a ‘patient-centred’operationalisation of the new dynamic concept of health: A mixed methods study. BMJ Open.

[22] Clement R.C., Welander A., Stowell C., Cha T.D., Chen J.L., Davies M., Fairbank J.C., Foley K.T., Gehrchen M., Hagg O. (2015). A proposed set of metrics for standardized outcome reporting in the management of low back pain. Acta Orthop..

[23] Chiarotto A., Deyo R.A., Terwee C.B., Boers M., Buchbinder R., Corbin T.P., Costa L.O.P., Foster N.E., Grotle M., Koes B.W. (2015). Core outcome domains for clinical trials in non-specific low back pain. Eur. Spine J..

[24] Sahin N., Karahan A.Y., Devrimsel G., Gezer İ.A. (2017). Comparison among pain, depression, and quality of life in cases with failed back surgery syndrome and non-specific chronic back pain. J. Phys. Ther. Sci..

[25] Turk D.C., Dworkin R.H., Allen R.R., Bellamy N., Brandenburg N., Carr D.B., Cleeland C., Dionne R., Farrar J.T., Galer B.S. (2003). Core outcome domains for chronic pain clinical trials: IMMPACT recommendations. Pain.

[26] Gorst S.L., Young B., Williamson P.R., Wilding J.P., Harman N.L. (2019). Incorporating patients’ perspectives into the initial stages of core outcome set development: A rapid review of qualitative studies of type 2 diabetes. BMJ Open Diabetes Res. Care.

[27] Noonan V.K., Lyddiatt A., Ware P., Jaglal S.B., Riopelle R.J., Bingham C.O., Figueiredo S., Sawatzky R., Santana M., Bartlett S.J. (2017). Montreal Accord on Patient-Reported Outcomes (PROs) use series e Paper 3: Patient-reported outcomes can facilitate shared decision-making and guide self-management. J. Clin. Epidemiol..

[28] Barry M.J., Edgman-Levitan S. (2012). Shared decision making—The pinnacle patient-centered care. N. Engl. J. Med..

[29] Epstein R.M., Peters E. (2009). Beyond information: Exploring patients’ preferences. JAMA.

[30] Kramer M.H.H., Bauer W., Dicker D., Durusu-Tanriover M., Ferreira F., Rigby S.P., Roux X., Schumm-Draeger P., Weidanz F., van Hulsteijn J. (2014). The changing face of internal medicine: Patient centred care. Eur. J. Intern. Med..

[31] Mühlbacher A.C., Juhnke C. (2013). Patient preferences versus physicians’ judgement: Does it make a difference in healthcare decision making?. Appl. Health Econ. Health Policy.

[32] Tricco A., Lillie E., Zarin W., O’Brien K.K., Colquhoun H., Levac D., Moher D., Peters M.D., Horsley T., Weeks L. (2018). PRISMA extension for scoping reviews (PRISMA-ScR): Checklist and explanation. Ann. Intern. Med..

[33] Ouzzani M., Hammady H., Fedorowicz Z., Elmagarmid A. (2016). Rayyan—A web and mobile app for systematic reviews. Syst. Rev..

[34] Braun V., Clarke V. (2006). Using thematic analysis in psychology. Qual. Res. Psychol..

[35] Lacey A., Luff D. (2009). Qualitative Research Analysis.

[36] Cieza A., Geyh S., Chatterji S., Kostanjsek N., Ustün B., Stucki G. (2005). ICF linking rules: An update based on lessons learned. J. Rehabil. Med..

[37] Cieza A., Fayed N., Bickenbach J., Prodinger B. (2019). Refinements of the ICF Linking Rules to strengthen their potential for establishing comparability of health information. Disabil. Rehabil..

[38] Guyatt G.H., Oxman A.D., Kunz R., Atkins D., Brozek J., Vist G., Alderson P., Glasziou P., Falck-Ytter Y., Schünemann H.J. (2011). GRADE guidelines: 2. Framing the question and deciding on important outcomes. J. Clin. Epidemiol..

[39] Diamond I.R., Grant R.C., Feldman B.M., Pencharz P.B., Ling S.C., Moore A.M., Wales P.W. (2014). Defining consensus: A systematic review recommends methodologic criteria for reporting of Delphi studies. J. Clin. Epidemiol..

[40] Munblit D., Nicholson T., Akrami A., Apfelbacher C., Chen J., De Groote W., Diaz J.V., Gorst S.L., Harman N., Kokorina A. (2022). A core outcome set for post-COVID-19 condition in adults for use in clinical practice and research: An international Delphi consensus study. Lancet Respir. Med..

[41] Gardner T., Refshauge K., McAuley J., Goodall S., Hübscher M., Smith L. (2015). Patient led goal setting in chronic low back pain—What goals are important to the patient and are they aligned to what we measure?. Patient Educ. Couns..

[42] O’Brien E.M., Staud R.M., Hassinger A.D., McCulloch R.C., Craggs J.G., Atchison J.T.W., Price D.D., Robinson M.E. (2010). Patient-centered perspective on treatment outcomes in chronic pain. Pain Med..

[43] Sanchez K., Papelard A., Nguyen C., Jousse M., Rannou F., Revel M., Poiraudeau S. (2009). Patient-preference disability assessment for disabling chronic low back pain: A cross-sectional survey. Spine.

[44] Sanchez K., Papelard A., Nguyen C., Bendeddouche I., Jousse M., Rannou F., Revel M., Poiraudeau S. (2011). McMaster-Toronto Arthritis Patient Preference Disability Questionnaire sensitivity to change in low back pain: Influence of shifts in priorities. PLoS ONE.

[45] Tinetti M.E., Costello D.M., Naik A.D., Davenport C., Hernandez-Bigos K., Van Liew J.R., Esterson J., Kiwak E., Dindo L. (2021). Outcome goals and health care preferences of older adults with multiple chronic conditions. JAMA Netw. Open.

[46] Moffett J.K., Torgerson D., Bell-Syer S., Jackson D., Llewlyn-Phillips H., Farrin A., Barber J. (1999). Randomised controlled trial of exercise for low back pain: Clinical outcomes, costs, and preferences. BMJ.

[47] Krabbe P.F., van Asselt A.D., Selivanova A., Jabrayilov R., Vermeulen K.M. (2019). Patient-centered item selection for a new preference-based generic health status instrument: CS-Base. Value Health.

[48] Tonkin K., Gustafsson L., Deen M., Broadbridge J. (2023). Multiple-Case Study Exploration of an Occupational Perspective in a Persistent Pain Clinic. Occup. Ther. J. Res..

[49] Karran E.L., Fryer C.E., Middleton J.T.W., Moseley G.L. (2022). Exploring the Social Determinants of Health Outcomes for Adults with Low Back Pain or Spinal Cord Injury and Persistent Pain: A Mixed Methods Study. J. Pain.

[50] Abbott A.D., Hedlund R., Tyni-lennÉ R. (2011). Patients’ experience post-lumbar fusion regarding back problems, recovery and expectations in terms of the international classification of functioning, disability and health. Disabil. Rehabil..

[51] Goudman L., Bruzzo A., van de Sande J., Moens M. (2020). Goal Identification Before Spinal Cord Stimulation: A Qualitative Exploration in Potential Candidates. Pain Pract..

[52] Hamm-Faber T.E., Engels Y., Vissers K.C.P., Henssen D. (2020). Views of patients suffering from Failed Back Surgery Syndrome on their health and their ability to adapt to daily life and self-management: A qualitative exploration. PLoS ONE.

[53] Ryan C.G., Eldabe S., Chadwick R., Jones S.E., Elliott-Button H.L., Brookes M., Martin D.J. (2019). An Exploration of the Experiences and Educational Needs of Patients With Failed Back Surgery Syndrome Receiving Spinal Cord Stimulation. Neuromodulation.

[54] Witkam R.L., Kurt E., van Dongen R., Arnts I., Steegers M.A.H., Vissers K.C.P., Henssen D., Engels Y. (2021). Experiences From the Patient Perspective on Spinal Cord Stimulation for Failed Back Surgery Syndrome: A Qualitatively Driven Mixed Method Analysis. Neuromodulation.

[55] Thomson S., Jacques L. (2009). Demographic characteristics of patients with severe neuropathic pain secondary to failed back surgery syndrome. Pain Pract..

[56] Alves Rodrigues T., de Oliveira E.J.S.G., Morais Costa B., Tajra Mualem Araújo R.L., Batista Santos Garcia J. (2022). Is There a Difference in Fear-Avoidance, Beliefs, Anxiety and Depression Between Post-Surgery and Non-Surgical Persistent Spinal Pain Syndrome Patients?. J. Pain Res..

[57] Santana M.J., Manalili K., Jolley R.J., Zelinsky S., Quan H., Lu M. (2018). How to practice person-centred care: A conceptual framework. Health Expect..

[58] Levy R.M., Mekhail N., Abd-Elsayed A., Abejón D., Anitescu M., Deer T.R., Eldabe S., Goudman L., Kallewaard J.T.W., Moens M. (2023). Holistic Treatment Response: An International Expert Panel Definition and Criteria for a New Paradigm in the Assessment of Clinical Outcomes of Spinal Cord Stimulation. Neuromodulation Technol. Neural Interface.

