# Supplementary material for: Clinical Patient-Relevant Outcome Domains for Persistent Spinal Pain Syndrome—A Scoping Review and Expert Panels
Source: J Clin Med. 2024 Mar 28;13(7):1975. doi: 10.3390/jcm13071975 (PMC11012536; doi:10.3390/jcm13071975)
Supplement: Supplementary file 1 [file jcm-13-01975-s001.zip › jcm-2893983-supplementary.pdf]

Journal of
Clinical Medicine

MDPI

Review

Supplementary file: Clinical Patient-Relevant Outcome Domains for Persistent Spinal Pain Syndrome—A Scoping Review and Expert Panels

Ferdinand Bastiaens [1,2,3,*], Jessica T. Wegener [3], Raymond W. J. G. Ostelo [4,5], Bert-Kristian W. P. van Roosendaal [2], Kris C. P. Vissers [2,3] and Miranda L. van Hooff [1,6]

1 Department of Research, Sint Maartenskliniek, 9500 GM Nijmegen, The Netherlands
2 Department of Anesthesiology, Pain, and Palliative Medicine, Radboud University Medical Center, Geert Grooteplein Zuid 10, 6525 GA Nijmegen, The Netherlands
3 Department of Anesthesiology and Pain Medicine, Sint Maartenskliniek, 9500 GM Nijmegen, The Netherlands
4 Department of Health Sciences, Faculty of Science and Amsterdam Movement Science Research Institute, Vrije Universiteit, Van der Boechorststraat 7, 1081 BT Amsterdam, The Netherlands
5 Department of Epidemiology and Data Science, Amsterdam University Medical Centre, Vrije Universiteit, Meibergdreef 9, 1105 AZ Amsterdam, The Netherlands
6 Department of Orthopedics, Radboud University Medical Center, Geert Grooteplein Zuid 10, 6525 GA Nijmegen, The Netherlands
* Correspondence: f.bastiaens@maartenskliniek.nl

Section S1: Search string (PubMed)

P:

(("Back Pain"[Mesh] OR "Lower Extremity"[Mesh] OR "Sciatica"[Mesh] OR “Leg pain”[tiab] OR “back pain”[tiab] OR laminectomy[tiab] OR “lower extremity”[tiab] OR Arachnoiditis*[tiab] OR Sciatic*[tiab]) AND ("Chronic Pain"[Mesh] OR "Radiculopathy"[Mesh] OR Radiculopath*[tiab] OR Neuropath*[tiab] OR radicular*[tiab] OR chronic[tiab])) OR (“Failed Back Surgery Syndrome”[tiab] OR FBSS[tiab] OR “Persistent Spinal Pain Syndrome”[tiab] OR PSPS[tiab] OR “Chronic pain after spinal surgery”[tiab] OR CPSS[tiab])

I:

"Pain Management"[MeSH] OR "Spinal Cord Stimulation"[MeSH] OR "Rehabilitation"[MeSH] OR pain management [tiab] OR spinal cord stimulation [tiab] OR rehabilitation [tiab] OR "invasive pain treatment*"[tiab] OR "pain surger*"[tiab]

O:

("Outcome Assessment, Health Care"[Mesh] OR "Patient Reported Outcome Measures"[Mesh] OR outcome* [tiab]) AND (Patient Satisfaction [MesH] OR Preference* [tiab] OR Attribute* [tiab] OR Experienc*[tiab] OR View* [tiab] OR “Choice behavior*” [tiab] OR “Choice behaviour*” [tiab] OR Motivation* [tiab] OR “Patient satisfaction”[tiab] OR “Patient selection”[tiab] OR “Patient participation”[tiab])

[19]

Table S1. Search results from PubMed.

Search number	Query	Results
4	1 AND 2 AND 3	1,037
3	"(""Outcome Assessment, Health Care""[Mesh] OR ""Patient Reported Outcome Measures""[Mesh] OR outcome* [tiab]) AND (Patient Satisfaction [MesH] OR Preference* [tiab] OR Attribute* [tiab] OR Experienc*[tiab] OR View* [tiab] OR ""Choice behavior*"" [tiab] OR ""Choice behaviour*"" [tiab] OR Motivation* [tiab] OR ""Patient satisfaction""[tiab] OR ""Patient selection""[tiab] OR ""Patient participation""[tiab])"	519,806
2	"""Pain Management""[MeSH] OR ""Spinal Cord Stimulation""[MeSH] OR ""Rehabilitation""[MeSH] OR pain management [tiab] OR spinal cord stimulation [tiab] OR rehabilitation [tiab] OR ""invasive pain treatment*""[tiab] OR ""pain surger*""[tiab]"	564,833
1	"((""Back Pain""[Mesh] OR ""Lower Extremity""[Mesh] OR ""Sciatica""[Mesh] OR ""Leg pain""[tiab] OR ""back pain""[tiab] OR laminectomy[tiab] OR ""lower extremity""[tiab] OR Arachnoiditis*[tiab] OR Sciatic*[tiab]) AND (""Chronic Pain""[Mesh] OR ""Radiculopathy""[Mesh] OR Radiculopath*[tiab] OR Neuropath*[tiab] OR radicular*[tiab] OR chronic[tiab])) OR (""Failed Back Surgery Syndrome""[tiab] OR FBSS[tiab] OR ""Persistent Spinal Pain Syndrome""[tiab] OR PSPS[tiab] OR ""Chronic pain after spinal surgery""[tiab] OR CPSS[tiab])"	51,933

Section S2: Qualitative overview of outcome domains from included studies.

Table S2. Qualitative overview of outcome domains from included studies.

Outcome domains	Abbot et al.	Goudman et al.	Hamm-Faber et al.	Ryan et al.	Witkam et al.
**Acceptance**			"Acceptance of chronic pain and associated limitations was a complicated process for all participants and lasted several years."		"According to the presurgery interviews, eight patients believed that SCS helped them in accepting their situation and enabled them to deal with the constraints coming with FBSS."
**Coping strategy**	"An additional six concepts were related to personal factors associated with coping style, beliefs, and locus		"All participants considered it essential to remain positive and put things in perspective in their current life		"Eleven patients disclosed an improved mental and emotional state of mind as a positive contributor to

	of control, while another two were not definable physical health concepts."		and to focus on the things they still can do despite their chronic pain."		their outcome, mostly as a result of reductions in analgesic intake and decreased pain levels. The latter coincides with the preoperative disclosures as patients expected that only a slight decrease of pain levels could enhance enjoyment of life."
**Daily activities**	"The most frequently linked categories included lifting and carrying objects (d430), acquisition of goods and services (d620), preparing meals (d630), doing house work (d640) and caring for household objects (d650)."		"Two interviewees mentioned fear when showering because the bending and lifting of the legs being painful, and they were afraid to fall. They wanted their partner to stay close to this action. Patients also mentioned difficulties dressing or putting on socks and taught themselves peculiar attitudes to be able to do this independently."	"Participants' expectations about SCS were not limited to pain relief and included reductions in medication, better sleep, and increased physical activity."	"Qualitatively, patients' improvements regarding chores and household were disparate between participants, ranging from no constraints at all to being unable to stand for longer than 5 min at the kitchen counter."
**Energy**	"The most frequent of all 24 body function categories linked were pain sensation (b280), emotional functions (b152), as well as energy and drive functions (b130)."		"All participants managed their time very carefully and weighed their activities based on the amount of energy and their rest moments. Therefore, they experienced that everything they		"They experienced being less tired during the day as a result."

			do, take twice as long."		
**Leisure Activities/ Hobbies**	"The most frequently linked categories included recreation and leisure (d950)."	"Being active and performing sports is the third participation goal ranked by 5 patients."	"Participants also mentioned the negative impact of chronic pain on leisure activities such as family outings or pursuing their hobby."		"While ten patients before surgery shared that they expected improvements, only eight of them mentioned that day trips became feasible again and that moments of leisure were experienced as more enjoyable."
**Mobility**	"The most frequently linked categories included changing and maintaining body position (d410–d415), walking and moving around (d450–d465), using transport (d470), and driving (d475)."	"In the domain of activities, walking (n = 15), sitting (n = 11), driving a car (n = 10), bending down (n = 11), and picking up (n = 9) were the highest ranked goals."	"They mentioned the growing immobility and impairments in walking, sitting, standing or bending and needed increasing moments of rest."	"There were several positive outcomes of SCS treatment reported, broadly in keeping with pre-treatment expectations/ wants, including pain relief, reductions in medication, better sleep, increased activity, and improved mood."	"Most patients experienced increased physical activity and mobility, either by foot or by bike, which coincided with their expectations."
**Mood**	"The most frequent of all 24 body function categories linked were pain sensation (b280), emotional functions (b152), as well as energy and drive functions (b130)."	"Eleven patients wanted to regain a feeling of happiness, and 5 patients wanted to focus on avoiding depression."		"There were several positive outcomes of SCS treatment reported, broadly in keeping with pretreatment expectations/ wants, including pain relief, reductions in medication, better sleep,	"Although all patients disclosed that FBSS influenced their state of mind negatively, only two expected SCS to improve their mood."

				increased activity, and improved mood."	
**Pain**	"The most frequent of all 24 body function categories linked were pain sensation (b280), emotional functions (b152), as well as energy and drive functions (b130)."	"In the domain of bodily functions and structure, all patients mentioned pain reduction."	"All participants stated that their low back and leg pain never completely disappeared after spinal surgery; instead, it increased over time and had changed their life."	"Participants' expectations about SCS were not limited to pain relief and included reductions in medication, better sleep, and increased physical activity."	"At the preoperative interview, all participants expected a decrease in pain intensity."
**Pain medication use**	"Furthermore, the use of analgesics, walking aids, family support, social security systems, health care systems, and labour market employment services were described as important environmental factors."		"Nevertheless, all participants mentioned that despite the pain medication, they were not pain-free, but felt limited and had to monitor their boundaries. Additionally, participants mentioned an increasing dosage to reach the same analgesic effect. They also mentioned all kind of side effects, such as decreased appetite, nausea, vomiting, drowsiness, fatigue and loss of concentration"	"Participants' expectations about SCS were not limited to pain relief and included reductions in medication, better sleep, and increased physical activity."	"In line with the preoperative expectations, all participants disclosed to have managed to either reduce or wean off their pain medication intake. Additionally, the medication induced side-effects, and a reduced level of awareness and energy in particular seemed to be the primary motivators."
**Perspective of life**			"The meaning of life for all interviewees was associated with doing something useful in family		"Additionally, three patients reported an altered life perspective

			life or society; however, they struggled with the physical and psychological shortcomings.”		and believed that it aids in enhancing their enjoyment of life. According to them, it helps to look on the bright side of life.”
**Self-reliance**			“Interviewees wished to maintain their independence as long as possible and indicated not quickly asking for help because this felt as shame, loss of control or loss of self-esteem.”		“Four patients reported that SCS enabled them to drive their car again, which increased their sense of independence. Baseline qualitative data showed that this was an expected outcome of SCS.”
**Sleep**			“Participants experienced low quality of sleep. They often awoke and experienced a restless sleep, i.e. short sleep periods and a lot of twisting and turning during the night, that caused them the feeling of insufficient rest on waking up in the morning.”	“Participants’ expectations about SCS were not limited to pain relief and included reductions in medication, better sleep, and increased physical activity.”	“Ten out of 11 implanted patients reported enhanced quality of sleep due to better sleep continuity, while only four of them explicitly expressed such expectations during the preoperative interview. They experienced being less tired during the day as a result.”
**Social participation**	“The most frequently linked categories included family relationships (d760).”	“Regaining a social life was the highest ranked goal for participation (n =	“In general, participants did not want to place their pain problem		“Preoperatively, all participants expected an increase of friends and relationships

		11), with the subthemes of making friends, seeing people, and spending more time with family.”	and disabilities at the centre of their family life; instead, they wanted to remain independent and tried to be meaningful in social life at a level they could handle.”		after SCS due to being able to be more socially active. Nonetheless, four patients still experienced a decline in their number of friends and social contacts.”
**Work**	“The most frequently linked categories included remunerative employment (d850).”	“Five patients indicated that return to work is one of their goals. The subthemes within this category are seeing people, being part of society, and financial motives.”	“All seven participants who had a paying job considered “paid work” and "staying at work" vital as they were meaningfully engaged and actively involved in society.”		“During the presurgery interview, five patients shared that they expected to return to work at least part-time, while no patients were employed prior to SCS.”

Section S3: Expert panel suggestions and discussion (translated from Dutch)

Table S3. Expert panel suggestions

**Chronic pain department**	
**Suggestions**	**Defined Outcome Domain**
“I understand my illness and have learned to live with it”	Part of acceptance and coping strategy
Experienced support	Merged with external perception
**Orthopedic department (spine unit)**	
External perception	External perception
Degree of secondary/disease gain	Secondary gain

Discussion:

- Exclusion of Work: Large proportion of PSPS patients are retired or incapacitated for work for a longer period of time, deemed important for younger patients with PSPS.
- Exclusion of Acceptance & Coping Strategy: Its importance was considered to depend on the phase of care the patients were in, which was more important in a later stage.
- As a clinician, it is difficult to say what patients really find important, as they do not indicate these things in such concrete terms.
- Pain medication use: Difficult to rate because the degree of pain medication is varies largely.
